# Blood zonulin levels in restless legs syndrome: insights into intestinal permeability

**DOI:** 10.1590/1806-9282.20241870

**Published:** 2025-08-08

**Authors:** Elif Sarica Darol, Elif Ozozen Sahin, Saadet Sayan, Murat Alemdar

**Affiliations:** 1 Sakarya University Training and Research Hospital, Department of Neurology – Serdivan, Türkiye.; 2 Sakarya University, Faculty of Medicine, Department of Medical Microbiology – Serdivan, Türkiye.; 3 Sakarya University, Faculty of Medicine, Department of Neurology – Serdivan, Türkiye.

**Keywords:** Restless leg syndrome, Intestinal permeability, Zonulin

## Abstract

**OBJECTIVE:**

Zonulin is a protein, synthesized in intestinal cells, that reversibly regulates the permeability of the intestinal epithelium. Elevated levels of zonulin are associated with increased intestinal permeability. It is shown that some neurological disorders are related to zonulin. We aim to research the possible associations between the zonulin levels and the symptom intensity in restless legs syndrome.

**METHOD:**

Demographic data of 43 patients with restless legs syndrome were recorded, and plasma zonulin levels were compared with 43 healthy controls using the enzyme-linked immunosorbent assay test. In the study, 66% of all participants were female and 20% were male. The mean age of the study group was 57.4±14.3 years. The average duration of the disease was 9.3±7.4 years, while the severity of the disease was 24.3±7.6. A p<0.05 was considered statistically significant.

**RESULTS:**

There was no significant difference between plasma zonulin levels in the control group and patients, but there were more individuals in the patient group in the high category of Zonulin levels, while the control group was dominant in the low level. In the patient group, zonulin levels were higher in the overweight and obese groups according to body mass index.

**CONCLUSION:**

Our study indirectly supports the hypothesis that central factors play a more significant role in the pathophysiology of restless legs syndrome rather than immunoresponsiveness in the gut and intestinal permeability.

## INTRODUCTION

Zonulin is a protein, synthesized in intestinal and liver cells, that reversibly regulates the permeability of the intestinal epithelium. It modulates the permeability of intercellular tight junctions (TJs) between cells of the wall of the digestive tract. Therefore, it plays a crucial role in maintaining the intestinal barrier function, which controls the passage of substances from the gut into the bloodstream. A tight control of permission through this barrier is essential to restrict the passage of harmful microorganisms and antigens to the bloodstream while allowing the absorption of nutrients and water at the same time. Plasma zonulin levels were measured as a marker of intestinal permeability. Elevated levels of zonulin are associated with increased intestinal permeability, commonly referred to as "leaky gut"^1,2^. This increased gut permeability has been associated with a variety of diseases, ranging from infections, metabolic diseases, and inflammatory and autoimmune diseases to neurological conditions^2,3^. The study by Fasano provides an overview of the role of zonulin in diseases characterized by inflammation and autoimmunity, which may be important for understanding restless leg syndrome (RLS)^2^.

Modulation of intestinal TJs by zonulin allows antigens and toxins to enter the bloodstream, triggering immune responses that can lead to several diseases in humans, in which autoimmunity plays the major role, such as celiac disease, type 1 diabetes mellitus (T1DM), multiple sclerosis (MS), rheumatoid arthritis, and inflammatory bowel diseases^4–6^. At the beginning of the twenty-first century, the leaky gut syndrome with subclinical inflammation is associated with T1DM and beta-cell autoimmunity^1^. Later, zonulin is revealed to be elevated with the digestion of gluten in patients with celiac disease, which in turn increases intestinal permeability. This allows gluten fragments to pass through the intestinal barrier and provoke an immune response. When gluten is removed from the diet, serum and fecal zonulin levels decrease, the intestine regains its native barrier function, and the autoantibody titers are normalized. Therefore, the autoimmune process shuts off, and, consequently, the intestinal damage begins to heal^4^.

Increased intestinal permeability mediated by zonulin has been linked to systemic inflammation, which can affect the central nervous system and contribute to the pathophysiology of its disorders. The role of zonulin in the gut-brain axis and its potential impact on neurological disorders has been a subject of increasing research interest. Zonulin is implicated in the regulation of the blood-brain barrier (BBB) in a similar way to its role in gut permeability. Elevated levels of zonulin may lead to increased BBB permeability, potentially contributing to neuroinflammation^5,6^. Serum zonulin level is revealed to be increased in patients with MS, in particular patients with progressive MS and relapsing-remitting MS with increased BBB permeability^6,7^. A study focused on the experimental autoimmune encephalomyelitis mouse model of MS has further described how zonulin is involved in MS. Intestinal permeability and intestinal zonulin are increased during the pre-clinical phase of neurological symptoms, suggesting a role for zonulin in disease development^8^.

In the context of zonulin and neurodegenerative diseases, there are several researches revealing elevated zonulin levels in diseases like Alzheimer's and Parkinson's^9,10^. Most probably, the increased zonulin levels might exacerbate the neuroinflammation, which then triggers the neurodegenerative cascades of those disorders.

RLS is a neurological disorder characterized by an uncontrollable urge to move one's legs, typically due to uncomfortable sensations. Telias and colleagues targeted the microbiota in the treatment of RLS, hypothesizing that increased intestinal permeability may lead to systemic inflammation that may contribute to RLS symptoms, and discussed the potential role of intestinal microbiota and permeability in RLS^11^. Chronic inflammation has also been suggested to play a role in the pathophysiology of RLS. A study by Trotti and Rye titled "Inflammation in restless legs syndrome: current status and future directions" examined the role of inflammation biomarkers influencing gut health and immunity in RLS patients, indicating that they may be indirectly important^12^.

To the best of our knowledge, there is no research directly linking zonulin and RLS. As previous studies suggest that gut permeability and inflammation, potentially regulated by zonulin, play a role in neurological disorders we conducted a clinical study to search for its possible role in RLS. We aim to research if there are any differences in the serum levels of zonulin between patients with RLS and healthy ones. In addition, we analyze the possible associations between the zonulin levels and the symptom intensity and obesity in RLS.

## METHODS

### Subjects

A total of 44 patients aged 18–85 years were admitted to Sakarya University Training and Research Hospital Outpatient Clinic who were diagnosed with RLS according to the International Restless Legs Syndrome Study (IRLSS) Group consensus criteria^13^ and had a duration of symptoms of at least 1 year and who could provide written consent were included in this study. Depending on the severity of symptoms, patients were divided into four subgroups using the IRLSS Group rating scale^14^. A total of 15 cases of our study group (34.09%) were newly diagnosed, and treatment had not been started yet; 20 of them (45%) were using only pramipexole therapy, 4 of them (9.09%) were using Fe replacement together with pramipexole therapy, and 5 of them (11%) were using gabapentinoid. In addition to RLS treatment, 7 (15.9%) of the patients were receiving vitamin B12 replacement, and 3 (6.8%) were receiving vitamin D replacement.

Patients with inflammatory bowel disorders (Crohn, ulcerative colitis, etc.), chronic renal failure, malignancy, and rheumatological disease were excluded. During the interview, all patients and controls were questioned about the presence of gastrointestinal symptoms such as diarrhea, and those who were detected were not included in the study. Age- and sex-matched volunteers from the staff of our clinic and their healthy relatives, in whom neurological disorders had been ruled out, conformed to our control group.

This study complies with the Declaration of Helsinki and was performed according to ethics committee approval. Written consent was obtained from all patients. The study was approved by the Ethics Committee of Sakarya University on 25.10.2023 with the number E-16214662-050.01.04.-305622-154.

### Blood samples

The demographic variables, medical history, and neurological examination findings were all noted by the senior author. Blood samples were centrifuged at 4,000 rpm for 10 min to separate the serum. The serums were aliquoted and stored in a −80° deep freezer until the day of the study. The kit and serums to be used on the day of the study were also brought to room temperature. Zonulin levels were measured by the enzyme-linked immunosorbent assay (ELISA) method using the kits from the Bioassay Technology Laboratory (Zhejiang, China). The in-measurement coefficient of variation (CV) of the kit was <8%, and the CV between measurements was <10%. The study was performed with the Triturus automated device (Grifols Diagnostic, Spain) in accordance with the company's recommendations. sST2 results were calculated quantitatively in ng/mL (signal/cut-off) units.

### Statistics

We used the Statistics Open For All package (released with the open-source AGPL3 license 2009–2014; Paton-Simpson and Associates Ltd, New Zealand) for the statistical analyses. A p<0.05 was considered statistically significant. In the descriptive statistics of the data, mean, standard deviation, median, min, max, frequency, and ratio values were used. The distribution of the variables was measured by the Kolmogorov-Smirnov test. After tests for normality, the statistical significance of the differences between the means of the groups was tested using an independent sample t-test for normally distributed data and the Mann-Whitney U-test for non-normally distributed data. Analysis of variance was used to test the possible differences between the averages of the obtained values in comparisons between multiple groups in normally distributed data, and the Kruskal-Wallis H-test for non-normally distributed data. The chi-square (χ^2^) test or Fisher's exact probability. The Pearson's linear correlation test was used for correlation analyses. Zonulin levels were divided into three groups (low, medium, and high) according to the thresholds determined using the pd.cut function, and mean and interquartile range (IQR) calculations were made.

## RESULTS

The study included 43 patients and 43 control individuals, comprising 66 women and 20 men. The mean age of the study group was 56.7±14.7 years, while the control group had a mean age of 51.6±14.6 years. There was no statistically significant difference between the groups in terms of mean age (p=0.1). A comparison of the sociodemographic and anthropometric data of the patient and control groups is presented in Table 1.

**Table 1:** Sociodemographic and anthropometric data of control and disease groups.

Characteristic		Control group (n=43)	Disease group (n=43)	p-value
Age (years)		51.6±14.6	56.7±14.7	0.10
Gender				0.30
	Female (%)	35 (81.4%)	31 (72.1%)	
	Male (%)	8 (18.6%)	12 (27.9%)	
	Height (cm)	165.3±8.1	167.3±8.2	0.20
	Weight (kg)	74.5±15.2	80.3±12.0	0.05
	BMI (kg/m^2^)	27.3±5.6	28.7±3.8	0.10
BMI categories				0.040
	<25 (%)	16 (37.2%)	6 (14.0%)	
	25–30 (%)	15 (34.9%)	20 (46.5%)	
	>30 (%)	12 (27.9%)	17 (39.5%)	
	Family history of RLS	0	26/17	–
	Disease duration (years)	–	9.3±7.4	–
	IRLSSG severity score	–	24.3±7.6	–

BMI: body mass index; RLS: restless leg syndrome; IRLSSG: International Restless Legs Syndrome Study Group.

No statistically significant differences were observed between the study and control groups in magnesium, ferritin, vitamin D, or vitamin B12 levels. However, the disease group exhibited slightly lower ferritin, Fe, and magnesium levels compared to the control group. Zonulin levels also showed no statistically significant difference between the two groups (Table 2). In linear correlation analyses, no significant associations were found between zonulin levels and study parameters (p>0.05).

**Table 2:** Laboratory data of control and disease groups (including normal ranges).

Parameter	Normal range	Control group	Disease group	p-value
Zonulin (ng/mL)	Not established	28.1±17.0	28.8±17.4	0.80
Hemoglobin (g/dL)	12.0–15.5	13.7±1.7	13.2±1.7	0.20
Hematocrit (%)	36–46	41.4±4.4	40.3±4.3	0.20
Iron (Fe, μg/dL)	50–170	75.1±35.1	62.5±29.8	0.090
Ferritin (ng/mL)	15–150	34.1±32.8	27.7±22.8	0.30
Magnesium (mg/dL)	1.7–2.2	1.98±0.15	1.93±0.18	0.20
TSH (μIU/mL)	0.4–4.0	2.05±0.86	2.08±1.46	0.90
Vitamin B12 (pg/mL)	200–900	324.5±135.1	409.7±230.2	0.05
Vitamin D (ng/mL)	20–50	13.8±8.	14.8±6.7	0.60

TSH: thyroid-stimulating hormone.

**Figure 1:**
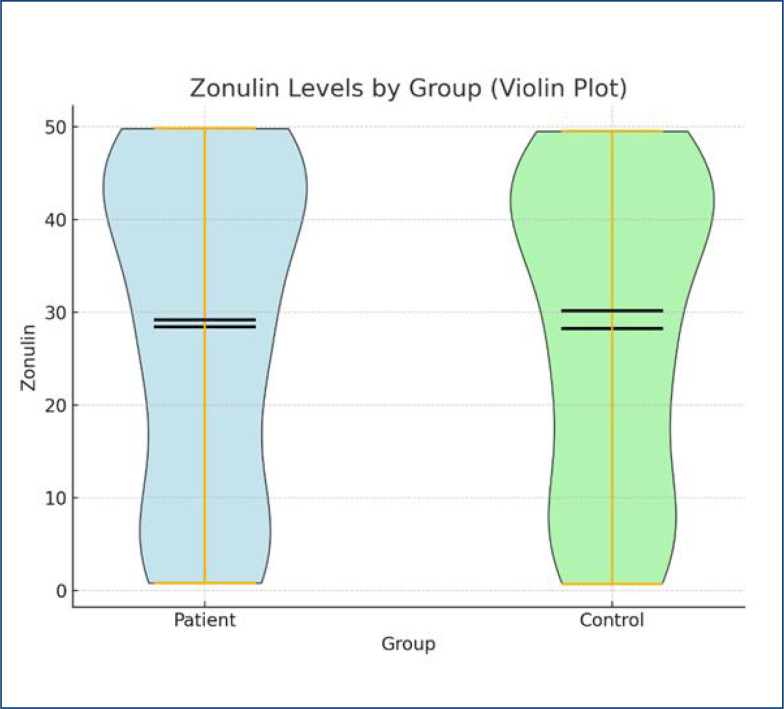
The distribution of median (IQR) zonulin levels in the patient and control groups on a violin plot. The median zonulin in patients: 28.3 ng/mL, and the median zonulin in controls: 28.7 ng/mL.

**Figure 2:**
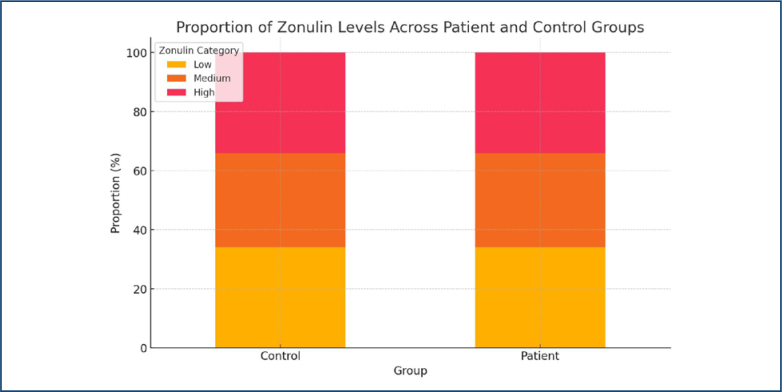
The stacked bar chart shows that a higher proportion of patients fall into the "High" Zonulin category, while the control group predominantly falls into the "Low" Zonulin category.

The median zonulin levels were similar in patients (28.39 ng/mL) and controls (28.78 ng/mL) (Figure 1). However, when zonulin levels were categorized, a greater proportion of individuals in the patient group fell into the "High" zonulin category, while the control group was more dominant in the "Low" category (Figure 2).

Body mass index (BMI) was slightly higher in the disease group (28.7 vs. 27.3), but this difference was not statistically significant (p=0.10). However, BMI categories showed a significant difference (p=0.04), with a higher proportion of individuals in the disease group having a BMI>30 (39.5 vs. 27.9%) (Table 1). Despite this, no statistically significant relationship was found between BMI categories and zonulin levels in either the patient or control groups (p=0.66 and p=0.89, respectively).

**Figure 3:**
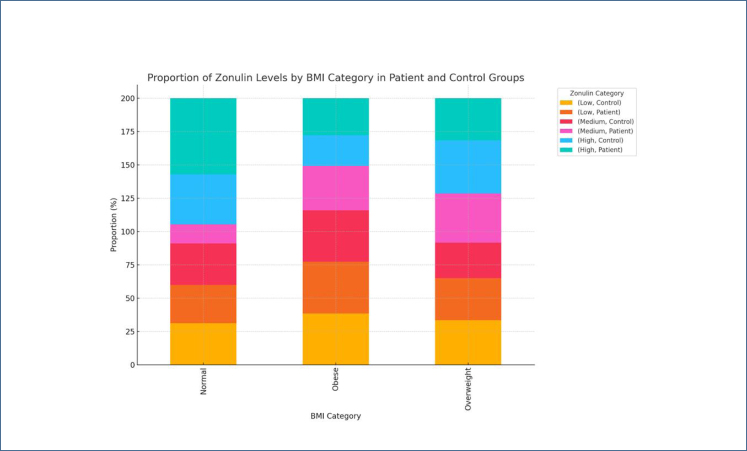
The distribution of zonulin levels according to body mass index categories in the patient and control groups. Zonulin levels tend to concentrate in the "High" category in overweight and obese patients.

**Figure 4:**
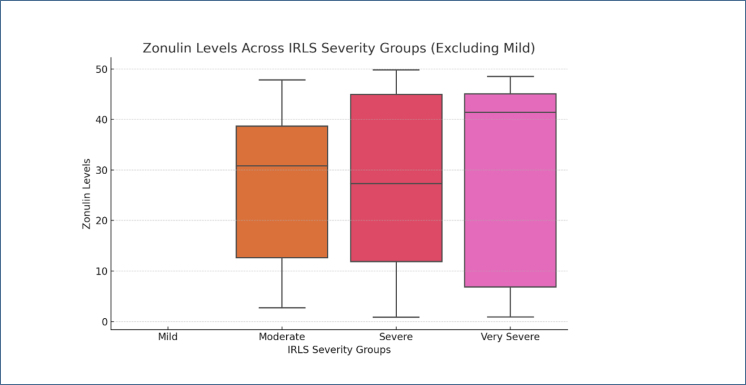
The distribution of International Restless Legs Syndrome Study severity and zonulin levels in the disease group. The intensity of high zonulin levels was noted in those with very severe International Restless Legs Syndrome Study (p>0.05).

When comparing zonulin levels across BMI categories within the patient group, it was observed that overweight and obese individuals tended to fall into the "High" zonulin category (Figure 3). Conversely, in the control group, zonulin levels were more concentrated in the "Low" and "Medium" categories, with no significant change in zonulin levels as BMI increased (Figure 3). There was no significant association between zonulin levels and disease severity (p=0.84). However, higher concentrations of zonulin were observed in individuals with very severe IRLS (Figure 4).

## DISCUSSION

The gut-brain axis, which encompasses bidirectional communication between the gastrointestinal tract and the nervous system, is increasingly recognized as a factor in the pathogenesis of various neurological disorders, including RLS. Previous research has focused on intestinal permeability and neuroinflammation, particularly in conditions such as Parkinson's disease. Proteins like zonulin, known to regulate intestinal permeability and the BBB through their action on the endothelial growth factor receptor, have been linked to disruptions in gastrointestinal barrier function. These disruptions allow macromolecules, including endotoxins, to cross the barrier and trigger immune responses^2,15,16^.

To the best of our knowledge, this study is the first to evaluate serum zonulin levels in primary RLS and to explore its relationship with intestinal permeability. RLS has been associated with inflammation and intestinal permeability in the context of other systemic disorders. Therefore, examining zonulin in this population may provide valuable insights.

Our case-control study demonstrated no significant difference in serum zonulin levels between patients with primary RLS and healthy controls. Furthermore, no correlation was found between zonulin levels and disease duration in RLS. Similarly, studies on idiopathic Parkinson's disease—a condition also linked to central dopaminergic deficiency—reported no relationship between zonulin levels and disease duration, although zonulin levels were elevated in idiopathic Parkinson's patients compared to controls^17^.

RLS is twice as common in women as in men and begins at the age of 40; the incidence increases with the age of 50 years^18^. Primary RLS can turn into a chronic progressive form with age. In a cross-sectional study conducted in Turkey, the prevalence of RLS in the population was reported to be 12.1%, reaching its highest level at 15.3% in the 50–65 age group. The same study associated the high prevalence of RLS among adults with the condition being generally unrecognized and a low rate of seeking professional medical help^19^. The participants in our study were older adults with an average age of 56 years, and most of them were long-term progressive RLS patients. Notably, approximately 35% of participants were drug-naïve, while the remainder were undergoing treatment for primary RLS. Medication use may have influenced biochemical results, a limitation acknowledged in our study.

It is known that dietary iron is converted to Fe^2+^ in the duodenum, transported into enterocytes, absorbed from the intestine, and stored in ferritin^20^. Our findings align with the established notion that central iron deficiency, rather than peripheral malabsorption of iron, underlies RLS pathogenesis^21^. Normal zonulin levels support prior research suggesting that the pathogenesis of RLS involves disruptions in the central nervous system's utilization of iron rather than intestinal permeability caused by a "leaky gut"^22^.

Interestingly, when zonulin levels were stratified based on obesity and RLS severity, higher zonulin levels were observed in obese participants compared to healthy controls. This suggests that obesity, rather than RLS, may drive zonulin elevation in this cohort. Consistent with our results, studies have demonstrated elevated zonulin levels in conditions such as obesity, obesity-related insulin resistance, type 1 and type 2 diabetes, and irritable bowel syndrome^21,23,24^. While these conditions are often associated with secondary RLS, our study focused exclusively on primary RLS cases, reinforcing the hypothesis that obesity may independently influence zonulin levels. Previous research has also reported a positive correlation between zonulin and BMI, which aligns with our findings^25,26^.

It is important to consider potential technical limitations associated with the ELISA kits used for measuring zonulin levels. Some studies have questioned the accuracy of commercially available zonulin assays and highlighted the need for functional validation^27^. Galipeau and Verdu emphasized that changes in TJ proteins, such as zonulin, must be corroborated with functional tests or RNA data to reliably assess intestinal permeability^28^. Fasano and Ajamian also highlighted inconsistencies in available ELISA kits and advised caution when interpreting zonulin measurements until standardized methodologies are developed^29,30^.

### Limitation of the study

When interpreting our findings, several limitations should be considered. First, the relatively small sample size limits the generalizability of our results to the broader population. Second, a significant limitation of this study is the absence of gut microbiota evaluations in both RLS patients and control subjects. Exploring the gut microbiota could have provided deeper insights into the relationship between intestinal permeability and RLS.

The cross-sectional design of the study also poses an important limitation, as it prevents us from establishing any causal relationships for the observed findings. Furthermore, we did not assess key factors such as socioeconomic status, dietary habits, and other psychosocial variables, which may influence serum zonulin levels and intestinal permeability. Individual differences, including genetic and environmental factors, may also affect intestinal permeability but were not evaluated in this study.

Finally, our patient group included individuals who were not exclusively untreated. Some patients were receiving supplementation, including vitamin B12 and vitamin D, which may have influenced the biochemical parameters assessed in our study. These factors should be considered when interpreting the results.

## CONCLUSION

Our study found no significant difference in zonulin levels between primary RLS patients and healthy controls. While obesity appears to be a confounding factor in zonulin elevation, normal zonulin levels in RLS support the hypothesis that central, rather than peripheral, mechanisms drive RLS pathogenesis. Future research should address technical limitations in zonulin measurement and explore additional markers to better elucidate the role of intestinal permeability in neurological disorders like RLS.

## Data Availability

The datasets generated and/or analyzed during the current study are available from the corresponding author upon reasonable request.
